# Plasma α-synuclein domain profiles across α-synucleinopathies

**DOI:** 10.1093/braincomms/fcaf189

**Published:** 2025-05-20

**Authors:** Marie-Laure Pons, Pablo Mohaupt, Jérôme Vialaret, Etienne Mondesert, Margaux Vignon, Salomé Coppens, Moreau Stéphane, Sylvain Lehmann, Christophe Hirtz

**Affiliations:** LBPC-PPC, Université de Montpellier, IRMB CHU de Montpellier, INM INSERM, Montpellier 34295, France; Shimadzu Corporation, LC-MS & Life Sciences, Duisburg 47269, Germany; LBPC-PPC, Université de Montpellier, IRMB CHU de Montpellier, INM INSERM, Montpellier 34295, France; LBPC-PPC, Université de Montpellier, IRMB CHU de Montpellier, INM INSERM, Montpellier 34295, France; LBPC-PPC, Université de Montpellier, IRMB CHU de Montpellier, INM INSERM, Montpellier 34295, France; LBPC-PPC, Université de Montpellier, IRMB CHU de Montpellier, INM INSERM, Montpellier 34295, France; LBPC-PPC, Université de Montpellier, IRMB CHU de Montpellier, INM INSERM, Montpellier 34295, France; Shimadzu Corporation, LC-MS & Life Sciences, Duisburg 47269, Germany; LBPC-PPC, Université de Montpellier, IRMB CHU de Montpellier, INM INSERM, Montpellier 34295, France; LBPC-PPC, Université de Montpellier, IRMB CHU de Montpellier, INM INSERM, Montpellier 34295, France

**Keywords:** α-synuclein, Parkinson’s disease, Lewy bodies, multiple system atrophy, mass spectrometry

## Abstract

The differential diagnosis of α-synucleinopathies, including Parkinson’s disease, dementia with Lewy bodies (DLB) and multiple system atrophy (MSA), remains challenging due to overlapping clinical features and the absence of reliable biomarkers. We developed a targeted mass spectrometry assay to profile α-synuclein peptides in plasma from Parkinson’s disease (*n* = 82), DLB (*n* = 32), MSA (*n* = 8) and controls (*n* = 21). We hypothesized that disease-specific truncations or post-translational modifications would alter levels of non-modified α-synuclein peptides across α-synucleinopathies. The assay quantified non-modified peptides derived from the N-terminus and non-amyloid component (NAC) domain, regions implicated in aggregate formation. Although peptide levels were consistent across disease groups, a distinct NAC domain pattern observed in MSA may reflect unique pathological processes. This study presents the first blood-based profiling of α-synuclein peptides in these disorders, offering a basis for further investigation into disease mechanisms. Refinement of the assay to include post-translational modifications could enhance understanding of α-synucleinopathies and support future biomarker development.

## Teaser

Pons *et al*. developed a mass spectrometry assay to profile α-synuclein peptides in plasma and assess differences across synucleinopathies. While we hypothesized that disease-specific truncations or modifications would alter non-modified peptide levels, no significant differences were observed. However, our findings provide valuable insights and establish a methodological foundation for future studies.

## Graphical Abstract

**Graphical Abstract:**
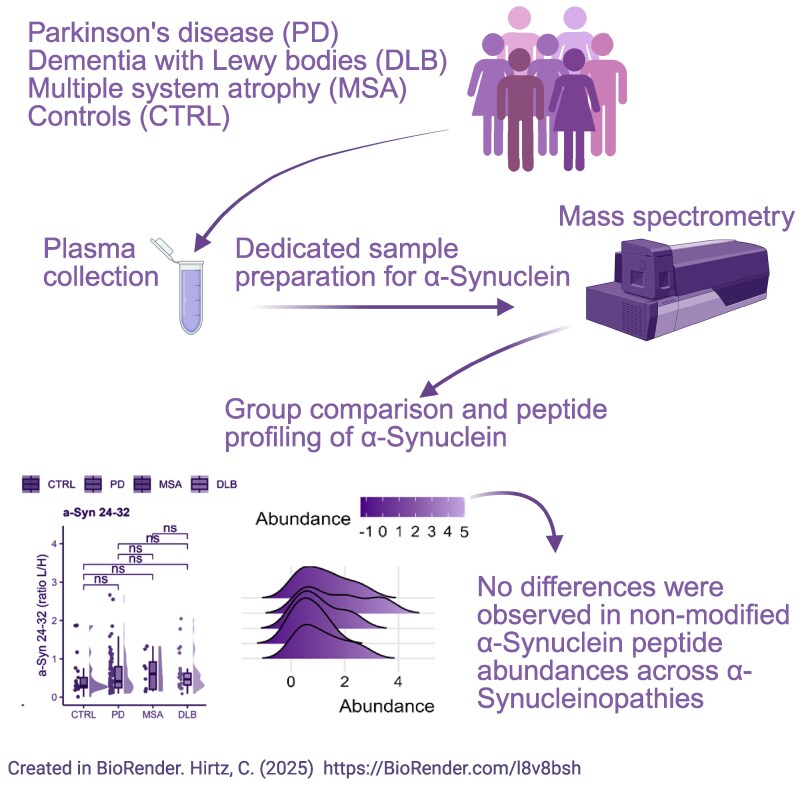

## Introduction

Parkinson's disease is a proteinopathy characterized by the misfolding and aggregation of α-synuclein into Lewy bodies and Lewy neurites in the brain parenchyma.^1^ Similar pathological features are observed in dementia with Lewy bodies (DLB) and multiple system atrophy (MSA), with occasional co-occurrence in Alzheimer’s disease.^2^ The primary diagnostic approach for these conditions remains clinical; DLB is diagnosed based on the McKeith criteria, which stipulate that cognitive dysfunction should precede or occur concurrently with motor dysfunction.^3^ In contrast, Parkinson’s disease typically manifests initially with motor symptoms and may later progress to cognitive deficits, termed Parkinson’s disease with dementia.^4^ MSA is uniquely characterized by α-synuclein aggregates in oligodendroglial cells and is diagnosed primarily through clinical symptoms and MRI findings that aid in differentiation from other α-synucleinopathies.^5^ The differential diagnosis among these disorders is hampered by overlapping clinical symptoms, particularly in the early stages of Parkinson’s disease and MSA. A key pathogenic mechanism in α-synucleinopathies, known as ‘seeding’, involves the recruitment of soluble α-synuclein monomers by misfolded α-synuclein fibrils, promoting their aggregation into neurotoxic amyloid structures.^6^ Recent advancements in seed amplification assays have shown promise in distinguishing these disorders by detecting distinct patterns of α-synuclein aggregation in cerebrospinal fluid (CSF).^7-12^ Further research suggests that the diagnostic value of these assays can be enhanced by combining them with measurements of neurofilament light chain.^13,14^ These developments indicate a potential shift from a purely clinical to a more biologically oriented diagnostic approach.^15^

With the advancement of disease-modifying therapies, accurate and efficient patient stratification is essential fortargeted treatments. The close resemblance among α-synucleinopathies necessitates biomarkers capable of detecting subtle differences in disease mechanisms and progression, thereby enabling precise patient classification and monitoring throughout therapeutic interventions. Efforts to use α-synuclein measurements for disease differentiation have been largely inconclusive, with plasma measurements considered of limited value due to the protein's extensive peripheral expression, particularly in erythrocytes.^16,17^ Nevertheless, parallels can be drawn with Tau protein in Alzheimer’s disease, which, despite its peripheral expression, reflects brain-specific pathological processes such as truncation and phosphorylation.^18-21^ This raises the possibility that α-synuclein could similarly serve as a marker of central nervous system pathology under appropriate conditions. Moreover, recent studies indicate that extracellular vesicles containing α-synuclein can cross the blood–brain barrier and enter the bloodstream, potentially allowing plasma α-synuclein to reflect CNS pathology.^22^

Emerging structural studies have revealed disease-specific conformations of pathological α-synuclein filaments. In Parkinson’s disease and DLB, the Lewy fold encompasses residues 31–100, spanning portions of the N-terminal, hydrophobic or non-amyloid component (NAC) and C-terminal regions.^23^ By contrast, MSA is characterized by distinct protofilaments.^24^ These structural differences potentially point to alternative truncation patterns or post-translational modifications and suggest unique pathological mechanisms that could be reflected in biofluid biomarkers.

We hypothesize that fluid biomarkers in α-synucleinopathies are shaped by disease-specific protease activity generating unique truncated forms or distinct post-translational modifications. For instance, α-synuclein is consistently phosphorylated at residue 129 across α-synucleinopathies, a modification detectable in skin biopsies with high diagnostic accuracy.^25^ This phosphorylation is present across all α-synucleinopathies, indicating it is not disease specific. In contrast, phosphorylation at amino acid residue 64 is unique to Parkinson’s disease and absent in MSA, suggesting it could differentiate these pathologies.^26^

To explore these possibilities, we developed a mass spectrometry-based method to measure peptides along the α-synuclein sequence in plasma. We hypothesized that disease-specific truncations or post-translational modifications would alter levels of non-modified α-synuclein peptides across α-synucleinopathies. This approach, while not directly targeting modifications, allows for the monitoring of non-modified peptides, which may provide indirect evidence of disease-specific truncation or modification.

## Materials and methods

### Study participants

Participants were recruited at the Montpellier Memory Resources Center. The study cohort included 143 individuals with clinically diagnosed Parkinson’s disease (*n* = 82), DLB (*n* = 32) and MSA (*n* = 8) and 21 control subjects with non-neurodegenerative conditions. These conditions included neurologic affections such as neuropathic (23.8%), vascular (28.6%), immunologic (28.6%) and hydrocephalus (19.0%). Diagnosis of Parkinson’s disease and MSA was established according to the International Parkinson and Movement Disorder Society criteria, while DLB was diagnosed based on the McKeith criteria.^3,27^ Plasma samples were collected and stored at the Montpellier Neurobank (CHU resource centre BB-0033-00031). Ethical approval was obtained from the Montpellier University Hospital’s regional Ethics Committee, and written informed consent was obtained from all participants. The demographic and clinical characteristics of participants are summarized in Table 1.

**Table 1:** Demographic characteristics of the patient cohorts

Demographic characteristic	Parkinson’s disease	DLB	MSA	Controls	*P*-value
*N*	82	32	8	21	
Age at sampling (years)	73.5 (67–77.75)	70 (66.75–75.33)	71.6 (66.65–76)	68 (65–74.7)	0.186
Sex (male/female)	50/32	27/5	3/5	16/5	<0.05
Disease duration (years)	7.5 (3–12) *n* = 78	3 (1–4) *n* = 31	4.2 (3–7.4)	1.8 (0.5–3)	<0.0001
Cognitive score (MMSE, MOCA or MDRS), %					
Positive, *n* (%)	25 (30.5)	3 (9.4)	4 (50)	2 (9.5)	
Negative, *n* (%)	14 (17.1)	28 (87.5)	3 (37.5)	11 (52.4)	
N/A, *n* (%)	43 (52.4)	1 (3.1)	1 (12.5)	8 (38.1)	
Hoehn and Yahr stage (UPDRS 5) %	Median Stage: 3 (32.9%), Main Range: Stages 2–3 (78%)	N/A	N/A	N/A	

Demographic characteristics are presented as medians and interquartile ranges. For categorical variables, differences across groups were evaluated using the *χ*^2^ goodness-of-fit test, and for continuous variables, the Kruskal–Wallis test was employed. The cognitive score is presented as positive or negative using cut-off values of 27 for MMSE, 24 for MOCA and 130 for MDRS. UPDRS, Unified Parkinson's disease rating scale.

### Sample preparation

EDTA plasma samples (95 µL) were thawed on ice for 1 h and diluted with 855 µL of deionized water. Samples were supplemented with 5.7 µL of 10 ng/µL recombinant full-length α-synuclein uniformly labelled with 15N (LGC, Teddington, UK) in 50 mM ammonium bicarbonate. After vortexing, 142.5 µL of 70% perchloric acid was added to precipitate non-target proteins, followed by a 15-min incubation on ice. The samples were centrifuged at 16 000 × *g* for 15 min at 4°C. Supernatants were transferred to LoBind tubes, and 95 µL of 1% trifluoroacetic acid was added. The samples were concentrated at room temperature using a vacuum concentrator (SpeedVac, Labconco). Solid-phase extraction was performed using RP-W tips on an AssayMap Bravo (Agilent Technologies). The tips were first equilibrated with water before loading the samples. After sample loading, the tips were washed with 10% acetonitrile containing 0.1% formic acid. The proteins of interest were then eluted with 45% acetonitrile containing 0.1% formic acid. The eluates were dried in a vacuum concentrator for 90 min at room temperature and reconstituted in 20 µL of 50 mM ammonium bicarbonate. Digestion was performed by adding 7 µL of 1 µg/µL Trypsin/LysC, followed by a 4-h incubation at 37°C with gentle agitation (450 rpm). Digestion was stopped by adding 0.5 µL of formic acid.

### Liquid chromatography-mass spectrometry multiple reaction monitoring analysis

Sample were analysed using a liquid chromatography system (Mikros, Shimadzu Corporation) coupled to an LCMS-8060 triple quadrupole mass spectrometer (Shimadzu Corporation) in positive ionization mode. Chromatographic separation was performed on a ZORBAX SB-Aq reversed-phase column (1 × 150 mm, particle size 3.5 µm, Agilent Technologies), at 35°C. The mobile phases were 0.1% formic acid in LCMS-grade water (Phase A) and 0.1% formic acid in acetonitrile (Phase B), with a 30-min gradient increasing Phase B from 0% to 30% at a flow rate of 50 µL/min. Multiple Reaction Monitoring method was employed with scheduled retention time windows. The ion source settings were optimized, including a nebulizing gas flow rate of 3 L/min, heating gas flow rate of 10 L/min, interface temperature of 300°C, desolvation line temperature of 250°C, heating block temperature of 400°C and drying gas flow rate of 10 L/min. Collision energy and dwell time were individually optimized for each peptide. Details on peptide positions and transitions are provided in Table 2.

**Table 2:** Selected α-synuclein peptides and their respective amino acid position, mass to charge ratio monitored in Q1 and fragment ions monitored in Q3

Acronym	Sequence	Amino acid position	Common with β-Syn	*m/z* Q1	*m/z* q3
α-Syn 1–6	(ac)MDVFMK	1–6	Yes	Not validated	
α-Syn 13–21	EGVVAAAEK	13–21	Yes	437.24	489.27 (y5) 588.34 (y6) 687.40 (y7)
α-Syn 24–32	QGVAEAAGK	24–32	No	415.72	546.29 (y6) 702.38 (y8) 684.33 (b8)
α-Syn 35–43	EGVLYVGSK	35–43	Yes	476.26	553.30 (y5) 666.38 (y6) 765.45 (y7) 822.47 (y8) 718.38 (b7)
α-Syn 46–58	EGVVHGVATVAEK	46–58	No	Not validated	
α-Syn 61–80	EQVTNVGGAVVTGVTAVAQK	61–80	No	643.35	773.45 (y8) 874.50 (y9) 973.57 (y10) 678.90 (y15)
α-Syn 81–96	TVEGAGSIAAATGFVK	81–96	No	739.90	551.32 (y5) 622.36 (y6) 693.39 (y7) 764.43 (y8) 1021.57 (y11)

Plasma QC samples with low (46.11 ng/mL), mid (74.70 ng/mL) and high (140.24 ng/mL) concentrations of α-synuclein were used for method validation. The validation included assessment of intra-assay coefficients of variation (CVs) at low (4.9–13%), mid (3.3–8%) and high (2.5–6%) concentrations and inter-assay CVs at low (12–22%), mid (13–16%) and high (10–14%) concentrations. Sample stability was evaluated on ice (6 h, recovery 88–103%), at room temperature (4 h, recovery 99–106%) and in the autosampler (48 h, recovery 95–121%). Dilution linearity was confirmed up to a 4-fold dilution with accuracy ranging from 87 to 117%, except for α-Syn 35–43 and α-Syn 81–96, which showed accurate results up to a 2-fold dilution (87–94%). Parallelism was tested to a 4-fold dilution with accuracy between 86 and 100%, except for α-Syn 61–80 and α-Syn 81–96, which were accurate to a 2-fold dilution (106–119%). The intra-assay CV during patient sample measurement, as determined using plasma QC sample, was 20.3–29.8% for α-synuclein-specific peptides and 18.7% and 40.0% for peptides shared between α-synuclein and β-synuclein, specifically α-Syn 13–21 and α-Syn 35–43, respectively.

### α-Synuclein quantification by commercially available immunoassay

Plasma samples were analysed using an immunochemiluminescence assay (MesoScale Discovery) to quantify total α-synuclein concentrations. Samples were diluted 200-fold prior to analysis, and all measurements were performed in accordance with the manufacturer’s instructions. The capture antibody was a rabbit monoclonal antibody targeting the C-terminal region of α-synuclein (amino acid residues 110–125), while the detection antibody was a mouse monoclonal antibody targeting amino acid residues 15–125.

### Data processing and statistical analysis

Data were processed using Skyline Software (version 20.1.0) and LabSolutions Insight Browser. Peaks were visually inspected and adjusted for accurate peak area calculations. Peptide abundances were quantified by comparing endogenous peptides with isotopically labelled standards.

Statistical analyses were performed in R (version 4.3.0). Homogeneity of variances was assessed with Levene’s test, and normality was evaluated using Shapiro–Wilk test, indicating deviations from normality on both raw and log-transformed residuals. Group comparisons were made using rank-based ANCOVA, adjusting for age and sex, followed by a *post hoc* pairwise analysis with Bonferroni correction. Peptide profiles were visualized using median abundance and ridgeline plots. Correlations between peptides were assessed with Spearman's rank correlation coefficients, visualized with correlograms. To assess the differential regulation among groups, receiver operating characteristic (ROC) curves were generated from logistic regression models that evaluated the contributions of peptides without incorporating confounders.

## Results

### Group-wise comparison at the peptide level for differential diagnosis in plasma

**Figure 1:**
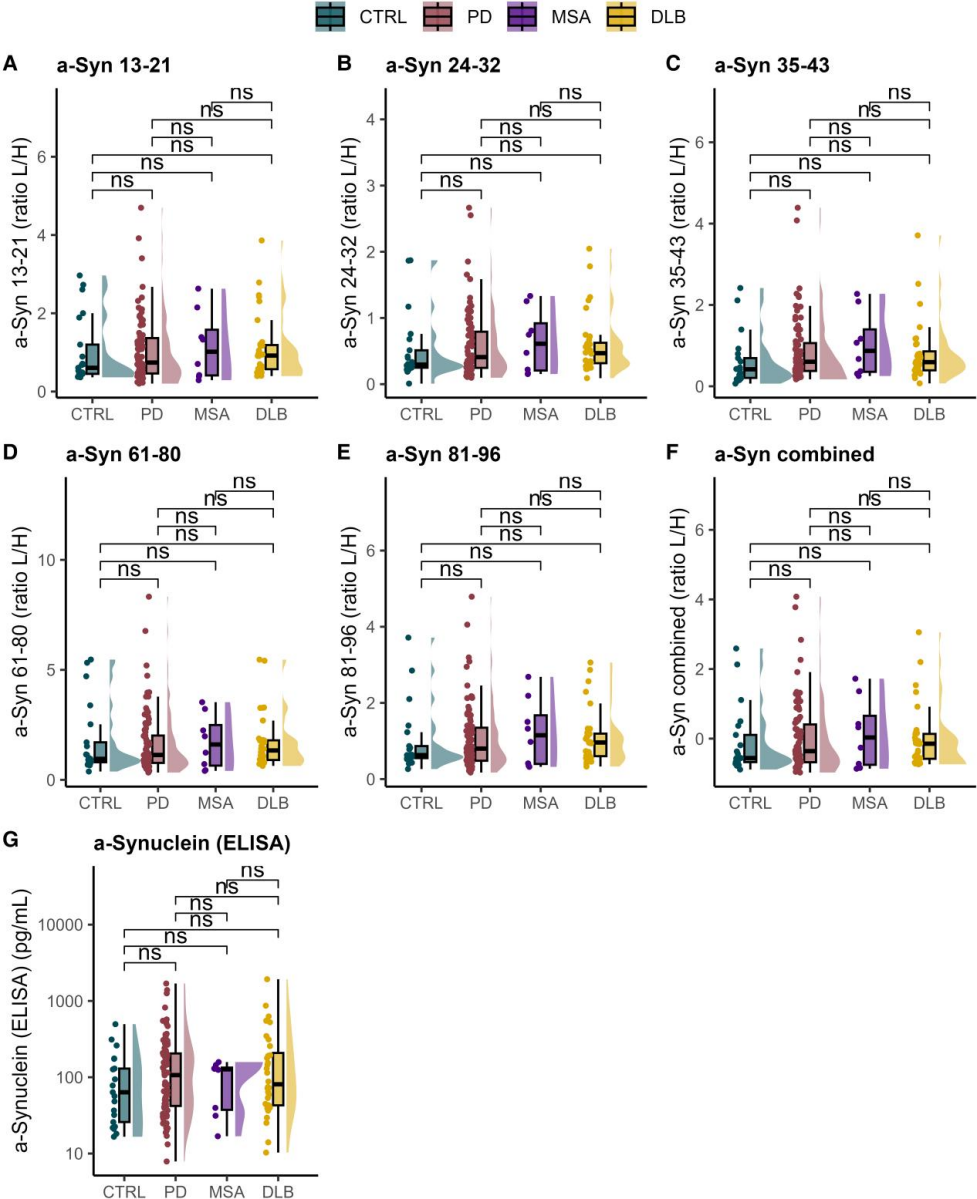
**Group-wise comparison of α-synuclein and corresponding peptide levels in clinical samples.** Individual plasma peptide levels from the N-terminal and NAC domains of α-synuclein: (**A**) α-Syn 13–21; (**B**) α-Syn 24–32; (**C**) α-Syn 35–43; (**D**) α-Syn 61–80; (**E**) α-Syn 81–96; (**F**) α-Syn combined; and (**G**) ELISA-based quantification of α-synuclein. Data are shown for individuals with DLB (*n* = 31), MSA (*n* = 8), Parkinson’s disease (*n* = 82) and CTRL (*n* = 21). The analysis includes individual boxplots for each peptide, a combined boxplot with mean *Z*-scored peptides representing total α-synuclein and a boxplot of ELISA results for α-synuclein. Statistical comparisons were performed using rank-based ANCOVA with age and sex as covariates, followed by a *post hoc* test with Bonferroni correction. No statistically significant effects of group were found for any variable (all *P* ≥ 0.425; *F* range: 0.03–0.94), and all *post hoc* pairwise tests were non-significant (*P* ≥ 0.05). The boxplots display the median, interquartile range and 1.5 interquartile range as a horizontal line, box and whiskers, respectively. The half violin plots illustrate data distribution. Each dot represents an individual sample measurement. The label ‘ns’ indicates a *P* > 0.05. α-Syn 13–21, α-synuclein amino acid residues 13–21; α-Syn 24–32, α-synuclein amino acid residues 24–32; α-Syn 35–43, α-synuclein amino acid residues 35–43; α-Syn 61–80, α-synuclein amino acid residues 61–80; α-Syn 81–96, α-synuclein amino acid residues 81–96; α-Syn combined, combined *Z*-scored peptide values representing total α-synuclein; ANCOVA, analysis of covariance; CTRL, controls; DLB, dementia with Lewy bodies; MSA, multiple system atrophy; ns, non-significant; PD, Parkinson’s disease; ratio L/H, the ratio of the light peptide and it’s isotopically labelled heavy form.

A group-wise comparison at the peptide level reveals similar profiles across conditions (Fig. 1). Specifically, our data suggest that α-synuclein levels may be marginally elevated in DLB compared with controls and slightly higher than in Parkinson’s disease. However, these differences were not statistically significant (*P* > 0.05). The modest elevation observed in MSA likely reflects the small sample size (*n* = 8) and also did not reach statistical significance (*P* > 0.05). The comparable levels of α-Syn 13–21 and α-Syn 35–43 with the other peptides suggest a limited impact of β-synuclein, given that α-Syn 13–21 and α-Syn 35–43 are also present in this protein. Plasma β-synuclein levels could not be included as a confounding factor due to the limited availability of blood-based assays for this protein.^28^ Enzyme-linked immunosorbent assay (ELISA) assay results, which measure total α-synuclein, did not show significant differences among groups. Furthermore, the concentrations measured by the ELISA vary widely among patient samples. This may be attributed to the choice of capture antibody that targets the C-terminus, an area known to undergo truncation and post-translational modification. There was no correlation observed between measurements and the Unified Parkinson's Disease Rating Scale (UPDRS) scores.

### Profiling plasma α-synuclein peptides in clinical samples

**Figure 2:**
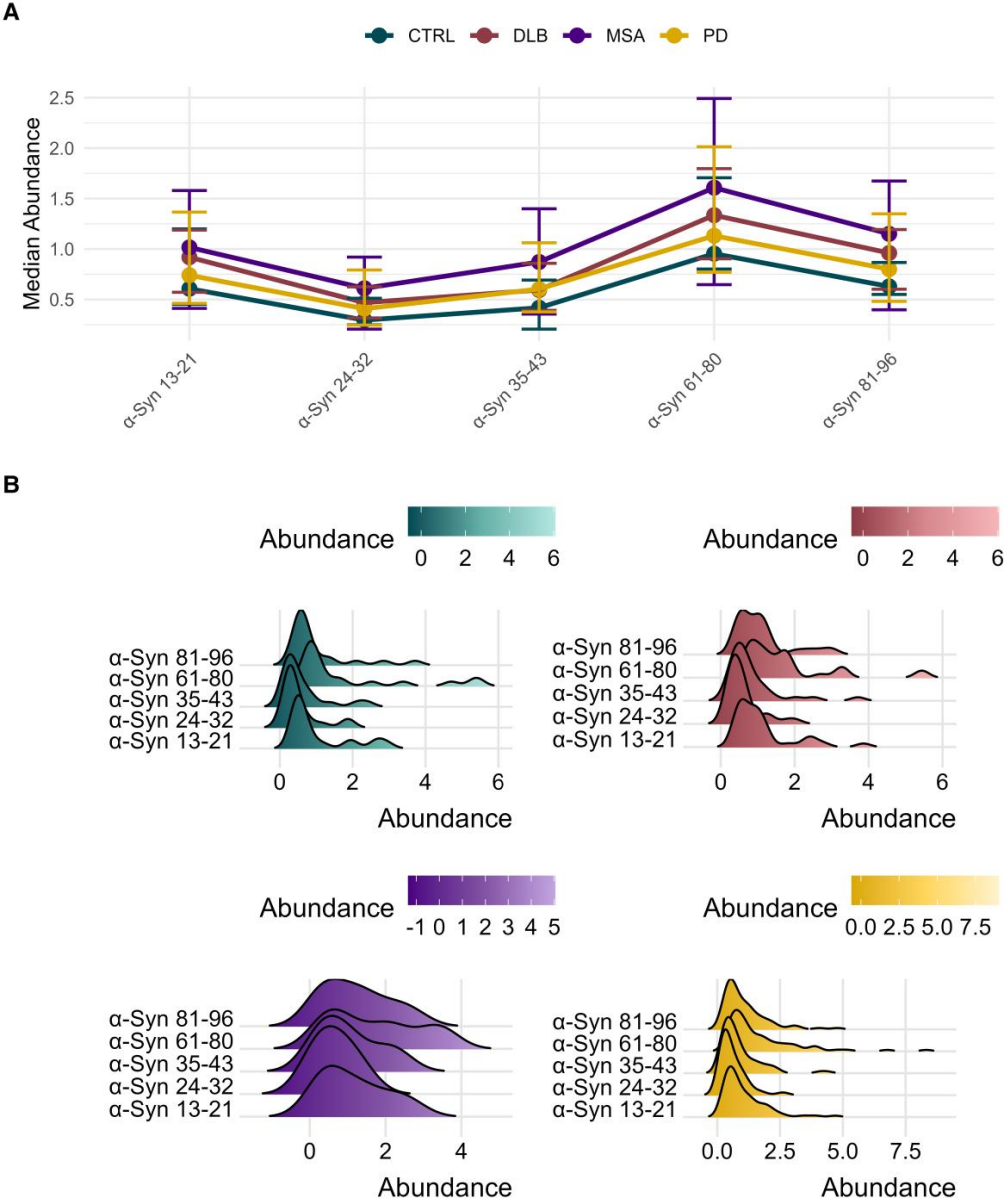
**Profiling plasma α-synuclein peptides in clinical samples.** (**A**) This figure displays the median abundance of α-synuclein peptides in plasma in different disease groups, Parkinson’s disease, CTRL, DLB and MSA. Each peptide is ordered along the *x*-axis by their amino acid position. The median abundances are depicted as coloured points, with error bars representing the interquartile ranges. The disease groups are colour coded: CTRL (teal), DLB (red), MSA (purple) and Parkinson’s disease (yellow). Lines connect the median values for each disease group across the different peptides to facilitate comparison. (**B**) The distribution of α-synuclein peptide abundances across the same disease groups. Each panel represents a different disease group, and within each panel, the abundance distributions of various peptides are shown using ridgeline plots. The peptides are ordered along the *y*-axis by their amino acid position. The ridgeline plots display the density of peptide abundances, with colours indicating the abundance levels.The *x*-axis represents the abundance levels, and the *y*-axis lists the peptides. No statistical tests were conducted for this figure. Median and interquartile ranges are shown to summarize the distribution of peptide abundance across disease groups. Abundance is shown as the ratio of light to heavy peptides and is therefore unitless. α-Syn 13–21, α-synuclein amino acid residues 13–21; α-Syn 24–32, α-synuclein amino acid residues 24–32; α-Syn 35–43, α-synuclein amino acid residues 35–43; α-Syn 61–80, α-synuclein amino acid residues 61–80; α-Syn 81–96, α-synuclein amino acid residues 81–96; CTRL, controls; DLB, dementia with Lewy bodies; MSA, multiple system atrophy; PD, Parkinson’s disease.

We analysed the peptide abundances across various disease groups to understand central tendencies and patterns (Fig. 2A). Our findings again indicate a slight increase in α-synuclein levels in α-synucleinopathies compared with controls. This increase is consistent along the entire N-terminus and NAC domain. Additionally, the patterns of peptide abundance are remarkably similar among the different disease groups, suggesting a conserved trend among these peptides. To further explore the distribution of peptide abundances within each disease group, we generated ridgeline plots (Fig. 2B). These plots provide a visual representation of the density distribution of peptide abundances, allowing for a detailed comparison of peptides within each disease group. While the ridgeline plots do not directly highlight increased α-synuclein levels, they reveal the overall distribution patterns and variability of peptide abundances across the disease groups, complementing the findings from the median abundance plot. The broader density distribution observed in MSA most likely represents the small group size.

### Associations between plasma α-synuclein peptides

**Figure 3:**
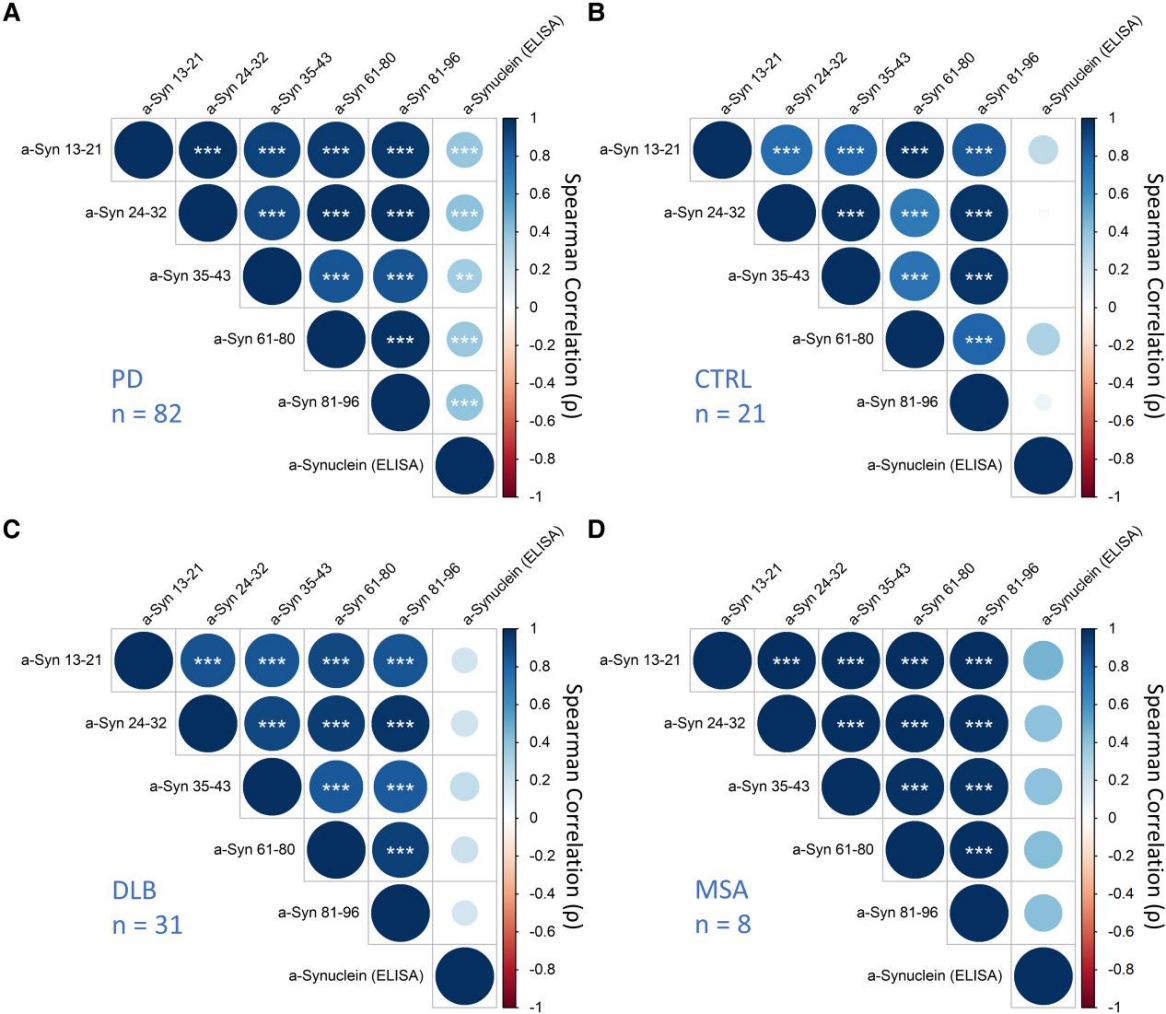
**Correlogram depicting α-synuclein peptide correlations in plasma within each diagnostic group.** The diagnostic groups include (**A**) Parkinson’s disease, (**B**) CTRL, (**C**) DLB and (**D**) MSA. The correlation matrices, visualized as correlograms, are based on Spearman's rank correlation coefficients. Circle size is proportional to the absolute value of the correlation coefficient, with larger circles indicating stronger correlations. Circle colour indicates the direction of the correlation. Darker shades represent stronger correlations. Significance levels are indicated as follows: *P ≤ 0.05, **P ≤ 0.01 and ***P ≤ 0.001. α-Syn 13–21, α-synuclein amino acid residues 13–21; α-Syn 24–32, α-synuclein amino acid residues 24–32; α-Syn 35–43, α-synuclein amino acid residues 35–43; α-Syn 61–80, α-synuclein amino acid residues 61–80; α-Syn 81–96, α-synuclein amino acid residues 81–96; CTRL, controls; DLB, dementia with Lewy bodies; MSA, multiple system atrophy; PD, Parkinson’s disease.

To assess whether peptide levels were interrelated within each diagnostic group, correlograms were utilized to visualize the associations (Fig. 3). These correlograms revealed strong correlations between peptides across all diagnostic groups, with Spearman’s rho ranging between 0.71 and 1.00, and *P* < 0.001. Associations with the ELISA were generally weak, with significant correlations only observed in the Parkinson’s disease group (*P* < 0.01 and *P* < 0.001), likely due to the larger sample size in this disease group. The positive correlations between peptides indicate a synchronous elevation across peptide levels. This finding, however, does not exclude the possibility of disproportionate regulation across different regions, as peptide levels can correlate while exhibiting disproportionate increases within a specific group.

### Exploring differential regulation through classification based on plasma α-synuclein peptide combinations using logistic regression

**Figure 4:**
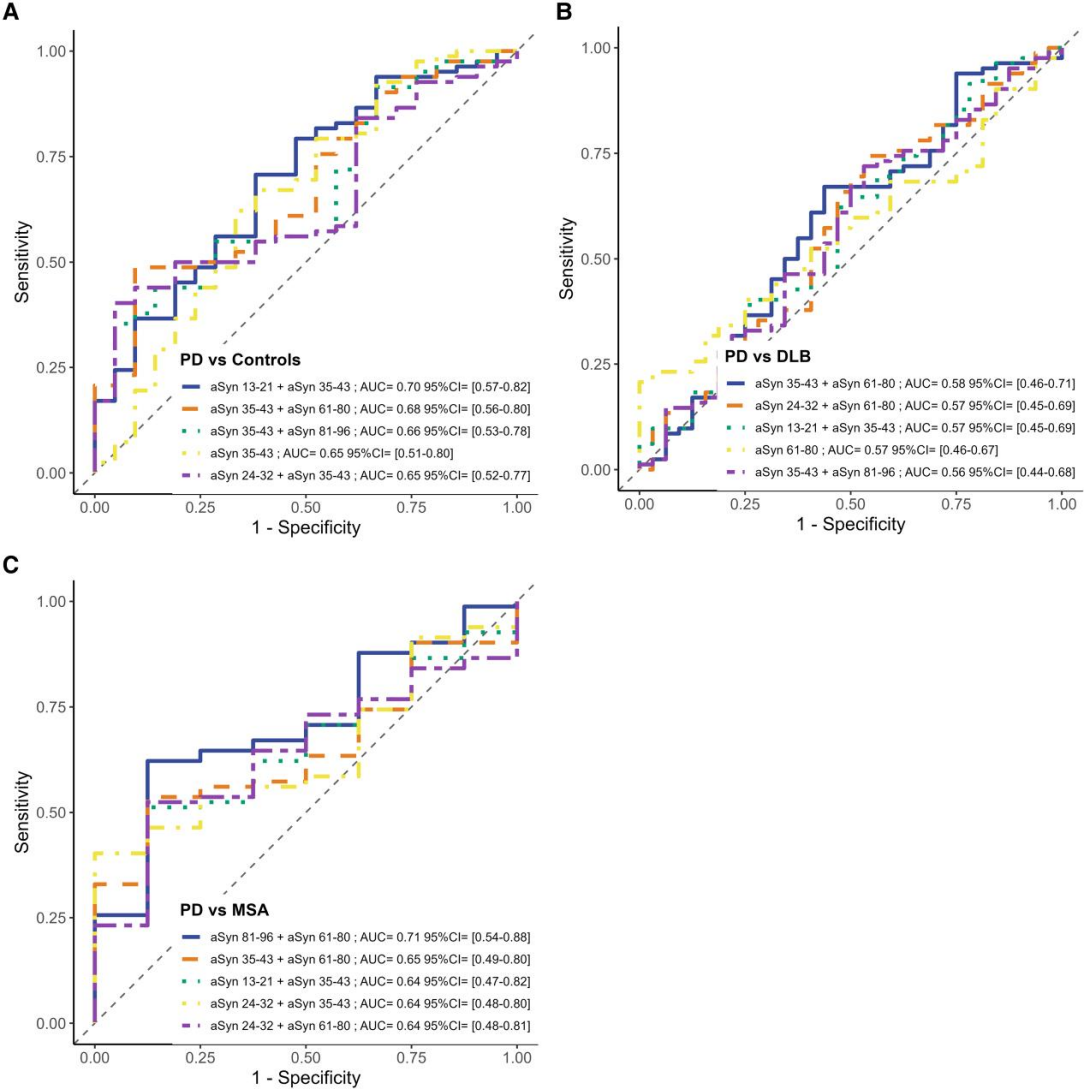
**ROC curves for classification based on α-synuclein peptide combinations.** ROC curves illustrate the diagnostic accuracy of individual peptides and peptide combinations derived from logistic regression models. The curves depict the ability to differentiate (**B**) Parkinson’s disease from dementia with Lewy bodies (DLB), (**C**) from multiple system atrophy (MSA) and (**A**) from control groups. Highlighted are the five peptides or peptide combinations with the highest AUC values, indicating the most significant diagnostic performance. α-Syn 13–21, α-synuclein amino acid residues 13–21; α-Syn 24–32, α-synuclein amino acid residues 24–32; α-Syn 35–43, α-synuclein amino acid residues 35–43; α-Syn 61–80, α-synuclein amino acid residues 61–80; α-Syn 81–96, α-synuclein amino acid residues 81–96; AUC, area under the curve; CTRL, controls; DLB, dementia with Lewy bodies; MSA, multiple system atrophy; PD, Parkinson’s disease.

To evaluate whether peptides were differentially regulated in specific groups, we conducted ROC analysis by creating peptide combinations using logistic regression models (Fig. 4; Supplementary Material 1). Notably, models including α-Syn 35–43 showed higher areas under the curve (AUCs). However, this is likely due to the high %CV during the cohort, but values are still provided for reference and interpretation. The resulting AUCs and Youden’s indices, as presented in Supplementary Table 1, do not directly indicate aberrant regulation among peptides. Specifically, no differences were observed between Parkinson’s disease and DLB, with AUCs not exceeding 0.60 and Youden’s indices < 0.25. Furthermore, Parkinson’s disease and DLB did not show discernible aberrations compared with control samples, with AUCs not exceeding 0.68 and Youden indices< 0.40. The MSA group showed potential dysregulation in the NAC domain, with combinations of α-Syn 61–80 and α-Syn 81–96 reaching AUCs of 0.71, 0.68 and 0.74 and Youden’s indices of 0.5, 0.44 and 0.54 against Parkinson’s disease, DLB and controls, respectively. Other combinations with α-Syn 61–80 also showed higher AUCs, potentially indicating some aberrant regulation of this peptide. However, it should be noted that this effect might be due to disproportionate group sizes.

## Discussion

In this study, we developed a novel mass spectrometry-based assay to monitor peptides from the N-terminus and NAC domain of α-synuclein in plasma, marking the first assessment of peptide levels from distinct regions of α-synuclein in blood. We hypothesized that disease-specific truncations or post-translational modifications would alter levels of non-modified α-synuclein peptides across α-synucleinopathies. However, no significant differences were observed between disease groups, suggesting that N-terminus and NAC domain profiles remain consistent, with slight elevations in α-synucleinopathies compared with controls. This uniformity may reflect shared mechanisms or indicate that disease-specific differences are subtle and not detected by non-modified peptide measurements.

α-Synucleinopathies share pathogenic features with tauopathies, such as the aggregation of misfolded proteins into toxic fibrils, suggesting that advances in Alzheimer’s disease diagnostics may inform biomarker development for similar proteinopathies. In Alzheimer’s disease, biomarkers like phosphorylation of tau at the 217th amino acid (pTau-217) highlight the role of post-translational modifications in distinguishing Alzheimer’s disease from other tauopathies, while N-terminal tau, linked to truncation, illustrates how targeting specific protein regions reflects disease mechanisms.^18-21,29,30^ The Aβ42/40 ratio further exemplifies this, capturing the differential cleavage of amyloid precursor protein to produce aggregation-prone Aβ42, whose reduction in biofluids serves as a key marker of amyloid pathology. These findings underscore the importance of monitoring specific protein regions and modifications to gain insights into disease mechanisms and guide the development of targeted biomarkers.

Our study explored whether similar principles observed in Alzheimer’s disease apply to α-synucleinopathies, focusing on potential disease-specific truncation or post-translational modifications of α-synuclein. Although we found no significant disproportional regulation at the peptide level, comparisons between Parkinson’s disease and MSA involving α-synuclein peptide 61–80 suggested aberrant regulation. This effect was not linked to known phosphorylation at the 64th amino acid residue in Parkinson’s disease, as it was not observed when comparing Parkinson’s disease with controls.^26^ The same pattern was observed in comparisons of MSA with DLB and controls. This peptide, part of the NAC region and identified as a key contributor to the formation of aggregates, suggests alternative regulatory mechanisms in MSA that may reflect unique pathological features.^31^ However, these findings require cautious interpretation due to small and disproportionate group sizes, which can affect the reliability and robustness of ROC analysis. Moreover, our focus on non-modified peptides does not exclude the possibility of differential regulation through disease-specific truncations or modifications occurring at levels that minimally affect non-modified peptide measurements.

This study contributes to the expanding research on α-synucleinopathies to improve diagnostic capabilities. There is an urgent clinical need for accessible biomarkers that can differentiate between Parkinson’s disease and MSA during the early stages of these conditions. As disease-modifying therapies advance, there remains a critical need for reliable methods to monitor disease progression. This need may not be adequately met by current seed amplification assays, highlighting the importance of developing new diagnostic biomarkers and a better understanding of α-synucleinopathies.

### Limitations

Our study employed an unconventional approach by investigating the disproportionate representation of peptides in plasma rather than CSF, which is preferred for such studies as it more accurately reflects brain pathologies *in vivo*. Therefore, effects observable in CSF may be missed or appear diluted in our results due to the peripheral expression of α-synuclein. Additionally, the small sample size of the MSA group and the disproportional group sizes could skew the results, particularly in ROC analysis. Consequently, any conclusions regarding MSA should be interpreted with caution and require validation in subsequent studies. Furthermore, these initial findings specifically monitor non-modified peptides, whereas a more comprehensive approach would also target their post-translationally modified forms. The increased %CVs during patient sample analysis also limit the accuracy of our outcomes. Nevertheless, the method is fit for purpose, as the primary goal of our study was to profile and discover disease-specific trends rather than to achieve absolute quantification.

## Conclusions

This study is the first to monitor the abundance of α-synuclein peptides from the N-terminus and NAC domain in plasma, revealing no detectable differential regulation of these domains among α-synucleinopathies via non-modified peptide measurements. Our findings provide a foundation for blood-based α-synuclein research, underscoring the need for assays that include post-translationally modified peptides to better understand the complexities of α-synucleinopathies.

## Supplementary Material

fcaf189_Supplementary_Data

## Data Availability

The datasets analysed during the current study are available from the corresponding author on reasonable request. The R scripts that are used are available in the Supplementary material.
