# Supplementary material for: Plasma α-synuclein domain profiles across α-synucleinopathies
Source: Brain Commun. 2025 May 20;7(3):fcaf189. doi: 10.1093/braincomms/fcaf189 (PMC12107063; doi:10.1093/braincomms/fcaf189)
Supplement: fcaf189_Supplementary_Data [file fcaf189_supplementary_data.docx]

**Supplementary material 1**

Contents

[**Supplementary Table 1.** Diagnostic accuracy of α-synuclein peptides and peptide combinations 1](#_Toc195297289)

[**R script Figure 1** 4](#_Toc195297290)

[**R script Figure 2** 8](#_Toc195297291)

[**R script Figure 3** 11](#_Toc195297292)

[**R script Figure 4** 13](#_Toc195297293)

# **Supplementary Table 1.** Diagnostic accuracy of α-synuclein peptides and peptide combinations

| PD vs. Controls | n | AUC | 95% CI | Sensitivity | Specificity | Youden’s Index |
| --- | --- | --- | --- | --- | --- | --- |
| α-Syn 13-21 | 81/21 | 0.52 | [0.38 – 0.66] | 0.51 | 0.67 | 0.18 |
| α-Syn 24-32 | 81/21 | 0.56 | [0.42 – 0.70] | 0.62 | 0.62 | 0.24 |
| α-Syn 35-43 | 81/21 | 0.65 | [0.51 – 0.80] | 0.67 | 0.62 | 0.29 |
| α-Syn 61-80 | 81/21 | 0.5 | [0.36 – 0.64] | 0.59 | 0.57 | 0.16 |
| α-Syn 81-96 | 81/21 | 0.54 | [0.41 – 0.67] | 0.6 | 0.62 | 0.22 |
| α-Syn 13-21 + α-Syn 24-32 | 81/21 | 0.54 | [0.40 – 0.68] | 0.34 | 0.86 | 0.2 |
| α-Syn 13-21 + α-Syn 35-43 | 81/21 | 0.7 | [0.57 – 0.82] | 0.71 | 0.62 | 0.33 |
| α-Syn 13-21 + α-Syn 61-80 | 81/21 | 0.58 | [0.45 – 0.71] | 0.49 | 0.81 | 0.3 |
| α-Syn 13-21 + α-Syn 81-96 | 81/21 | 0.51 | [0.37 – 0.65] | 0.59 | 0.52 | 0.11 |
| α-Syn 24-32 + α-Syn 35-43 | 81/21 | 0.65 | [0.52 – 0.77] | 0.4 | 0.95 | 0.35 |
| α-Syn 24-32 + α-Syn 61-80 | 81/21 | 0.58 | [0.45 – 0.72] | 0.55 | 0.67 | 0.22 |
| α-Syn 24-32 + α-Syn 81-96 | 81/21 | 0.57 | [0.44 – 0.71] | 0.49 | 0.76 | 0.25 |
| α-Syn 35-43 + α-Syn 61-80 | 81/21 | 0.68 | [0.56 – 0.80] | 0.49 | 0.9 | 0.39 |
| α-Syn 35-43 + α-Syn 81-96 | 81/21 | 0.66 | [0.53 – 0.78] | 0.35 | 0.95 | 0.31 |
| α-Syn 61-80 + α-Syn 81-96 | 81/21 | 0.57 | [0.43 – 0.70] | 0.34 | 0.86 | 0.2 |
| PD vs. DLB | **n** | **AUC** | **95% CI** | **Sensitivity** | **Specificity** | **Youden’s Index** |
| α-Syn 13-21 | 81/32 | 0.55 | [0.44 – 0.67] | 0.28 | 0.91 | 0.19 |
| α-Syn 24-32 | 81/32 | 0.52 | [0.41 – 0.64] | 0.21 | 0.97 | 0.18 |
| α-Syn 35-43 | 81/32 | 0.50 | [0.39 – 0.62] | 0.37 | 0.75 | 0.12 |
| α-Syn 61-80 | 81/32 | 0.57 | [0.46 – 0.67] | 0.21 | 1 | 0.21 |
| α-Syn 81-96 | 81/32 | 0.54 | [0.43 – 0.65] | 0.22 | 0.94 | 0.16 |
| α-Syn 13-21 + α-Syn 24-32 | 81/32 | 0.53 | [0.41 – 0.66] | 0.78 | 0.41 | 0.19 |
| α-Syn 13-21 + α-Syn 35-43 | 81/32 | 0.57 | [0.45 – 0.69] | 0.62 | 0.53 | 0.15 |
| α-Syn 13-21 + α-Syn 61-80 | 81/32 | 0.5 | [0.37 – 0.62] | 0.57 | 0.5 | 0.07 |
| α-Syn 13-21 + α-Syn 81-96 | 81/32 | 0.52 | [0.40 – 0.64] | 0.46 | 0.66 | 0.12 |
| α-Syn 24-32 + α-Syn 35-43 | 81/32 | 0.56 | [0.44 – 0.68] | 0.7 | 0.53 | 0.23 |
| α-Syn 24-32 + α-Syn 61-80 | 81/32 | 0.57 | [0.45 – 0.69] | 0.74 | 0.47 | 0.21 |
| α-Syn 24-32 + α-Syn 81-96 | 81/32 | 0.54 | [0.41 – 0.66] | 0.44 | 0.69 | 0.13 |
| α-Syn 35-43 + α-Syn 61-80 | 81/32 | 0.58 | [0.46 – 0.71] | 0.67 | 0.56 | 0.23 |
| α-Syn 35-43 + α-Syn 81-96 | 81/32 | 0.56 | [0.44 – 0.68] | 0.72 | 0.47 | 0.19 |
| α-Syn 61-80 + α-Syn 81-96 | 81/32 | 0.53 | [0.40 – 0.66] | 0.68 | 0.5 | 0.18 |
| PD vs. MSA | **n** | **AUC** | **95% CI** | **Sensitivity** | **Specificity** | **Youden’s Index** |
| α-Syn 13-21 | 81/8 | 0.54 | [0.29 – 0.79] | 0.74 | 0.5 | 0.24 |
| α-Syn 24-32 | 81/8 | 0.54 | [0.28 – 0.79] | 0.73 | 0.5 | 0.23 |
| α-Syn 35-43 | 81/8 | 0.57 | [0.34 – 0.81] | 0.74 | 0.5 | 0.24 |
| α-Syn 61-80 | 81/8 | 0.52 | [0.27 – 0.78] | 0.72 | 0.5 | 0.22 |
| α-Syn 81-96 | 81/8 | 0.54 | [0.27 – 0.80] | 0.74 | 0.5 | 0.24 |
| α-Syn 13-21 + α-Syn 24-32 | 81/8 | 0.61 | [0.39 – 0.82] | 0.78 | 0.5 | 0.28 |
| α-Syn 13-21 + α-Syn 35-43 | 81/8 | 0.64 | [0.47 – 0.82] | 0.51 | 0.88 | 0.39 |
| α-Syn 13-21 + α-Syn 61-80 | 81/8 | 0.61 | [0.41 – 0.80] | 0.49 | 0.75 | 0.24 |
| α-Syn 13-21 + α-Syn 81-96 | 81/8 | 0.55 | [0.30 – 0.81] | 0.71 | 0.62 | 0.33 |
| α-Syn 24-32 + α-Syn 35-43 | 81/8 | 0.64 | [0.48 – 0.80] | 0.4 | 1 | 0.4 |
| α-Syn 24-32 + α-Syn 61-80 | 81/8 | 0.64 | [0.48 – 0.81] | 0.52 | 0.88 | 0.4 |
| α-Syn 24-32 + α-Syn 81-96 | 81/8 | 0.6 | [0.42 – 0.78] | 0.38 | 1 | 0.38 |
| α-Syn 35-43 + α-Syn 61-80 | 81/8 | 0.65 | [0.49 – 0.80] | 0.54 | 0.88 | 0.41 |
| α-Syn 35-43 + α-Syn 81-96 | 81/8 | 0.58 | [0.35 – 0.81] | 0.76 | 0.5 | 0.26 |
| α-Syn 61-80 + α-Syn 81-96 | 81/8 | 0.71 | [0.54 – 0.88] | 0.62 | 0.88 | 0.5 |
| DLB vs. Controls | **n** | **AUC** | **95% CI** | **Sensitivity** | **Specificity** | **Youden’s Index** |
| α-Syn 13-21 | 32/21 | 0.6 | [0.43 – 0.77] | 0.81 | 0.48 | 0.29 |
| α-Syn 24-32 | 32/21 | 0.63 | [0.46 – 0.79] | 0.78 | 0.57 | 0.35 |
| α-Syn 35-43 | 32/21 | 0.66 | [0.50 – 0.82] | 0.88 | 0.48 | 0.35 |
| α-Syn 61-80 | 32/21 | 0.58 | [0.41 – 0.75] | 0.59 | 0.67 | 0.26 |
| α-Syn 81-96 | 32/21 | 0.6 | [0.44 – 0.76] | 0.56 | 0.76 | 0.32 |
| α-Syn 13-21 + α-Syn 24-32 | 32/21 | 0.64 | [0.47 – 0.80] | 0.75 | 0.62 | 0.37 |
| α-Syn 13-21 + α-Syn 35-43 | 32/21 | 0.63 | [0.47 – 0.79] | 0.81 | 0.48 | 0.29 |
| α-Syn 13-21 + α-Syn 61-80 | 32/21 | 0.57 | [0.42 – 0.73] | 0.44 | 0.81 | 0.25 |
| α-Syn 13-21 + α-Syn 81-96 | 32/21 | 0.6 | [0.43 – 0.76] | 0.56 | 0.76 | 0.32 |
| α-Syn 24-32 + α-Syn 35-43 | 32/21 | 0.62 | [0.46 – 0.77] | 0.41 | 0.9 | 0.31 |
| α-Syn 24-32 + α-Syn 61-80 | 32/21 | 0.59 | [0.43 – 0.75] | 0.62 | 0.67 | 0.29 |
| α-Syn 24-32 + α-Syn 81-96 | 32/21 | 0.55 | [0.39 – 0.72] | 0.56 | 0.67 | 0.23 |
| α-Syn 35-43 + α-Syn 61-80 | 32/21 | 0.42 | [0.26 – 0.58] | 0.16 | 0.9 | 0.06 |
| α-Syn 35-43 + α-Syn 81-96 | 32/21 | 0.6 | [0.44 – 0.75] | 0.38 | 0.9 | 0.28 |
| α-Syn 61-80 + α-Syn 81-96 | 32/21 | 0.55 | [0.39 – 0.71] | 0.44 | 0.76 | 0.2 |
| DLB vs. MSA | **n** | **AUC** | **95% CI** | **Sensitivity** | **Specificity** | **Youden’s Index** |
| α-Syn 13-21 | 32/8 | 0.49 | [0.20 – 0.78] | 0.81 | 0.5 | 0.31 |
| α-Syn 24-32 | 32/8 | 0.51 | [0.21 – 0.81] | 0.84 | 0.5 | 0.34 |
| α-Syn 35-43 | 32/8 | 0.55 | [0.28 – 0.83] | 0.81 | 0.5 | 0.31 |
| α-Syn 61-80 | 32/8 | 0.49 | [0.19 – 0.79] | 0.81 | 0.5 | 0.31 |
| α-Syn 81-96 | 32/8 | 0.52 | [0.22 – 0.81] | 0.81 | 0.5 | 0.31 |
| α-Syn 13-21 + α-Syn 24-32 | 32/8 | 0.51 | [0.24 – 0.77] | 0.69 | 0.62 | 0.31 |
| α-Syn 13-21 + α-Syn 35-43 | 32/8 | 0.68 | [0.51 – 0.85] | 0.53 | 0.88 | 0.41 |
| α-Syn 13-21 + α-Syn 61-80 | 32/8 | 0.61 | [0.41 – 0.81] | 0.28 | 1 | 0.28 |
| α-Syn 13-21 + α-Syn 81-96 | 32/8 | 0.6 | [0.34 – 0.86] | 0.88 | 0.5 | 0.38 |
| α-Syn 24-32 + α-Syn 35-43 | 32/8 | 0.67 | [0.49 – 0.84] | 0.56 | 0.88 | 0.44 |
| α-Syn 24-32 + α-Syn 61-80 | 32/8 | 0.69 | [0.51 – 0.86] | 0.59 | 0.88 | 0.47 |
| α-Syn 24-32 + α-Syn 81-96 | 32/8 | 0.58 | [0.33 – 0.83] | 0.78 | 0.5 | 0.28 |
| α-Syn 35-43 + α-Syn 61-80 | 32/8 | 0.7 | [0.53 – 0.87] | 0.66 | 0.88 | 0.53 |
| α-Syn 35-43 + α-Syn 81-96 | 32/8 | 0.67 | [0.48 – 0.86] | 0.5 | 0.88 | 0.38 |
| α-Syn 61-80 + α-Syn 81-96 | 32/8 | 0.68 | [0.49 – 0.87] | 0.56 | 0.88 | 0.44 |
| MSA vs. Controls | **n** | **AUC** | **95% CI** | **Sensitivity** | **Specificity** | **Youden’s Index** |
| α-Syn 13-21 | 8/21 | 0.51 | [0.23 – 0.80] | 0.5 | 0.76 | 0.26 |
| α-Syn 24-32 | 8/21 | 0.56 | [0.27 – 0.85] | 0.62 | 0.71 | 0.34 |
| α-Syn 35-43 | 8/21 | 0.69 | [0.48 – 0.90] | 0.5 | 0.86 | 0.36 |
| α-Syn 61-80 | 8/21 | 0.51 | [0.23 – 0.79] | 0.62 | 0.67 | 0.29 |
| α-Syn 81-96 | 8/21 | 0.55 | [0.25 – 0.85] | 0.62 | 0.76 | 0.39 |
| α-Syn 13-21 + α-Syn 24-32 | 8/21 | 0.57 | [0.27 – 0.86] | 0.62 | 0.81 | 0.43 |
| α-Syn 13-21 + α-Syn 35-43 | 8/21 | 0.9 | [0.78 – 1.00] | 1 | 0.67 | 0.67 |
| α-Syn 13-21 + α-Syn 61-80 | 8/21 | 0.74 | [0.54 – 0.95] | 0.75 | 0.71 | 0.46 |
| α-Syn 13-21 + α-Syn 81-96 | 8/21 | 0.6 | [0.31 – 0.88] | 0.62 | 0.86 | 0.48 |
| α-Syn 24-32 + α-Syn 35-43 | 8/21 | 0.88 | [0.76 – 1.00] | 1 | 0.71 | 0.71 |
| α-Syn 24-32 + α-Syn 61-80 | 8/21 | 0.78 | [0.59 – 0.97] | 0.75 | 0.86 | 0.61 |
| α-Syn 24-32 + α-Syn 81-96 | 8/21 | 0.55 | [0.25 – 0.85] | 0.62 | 0.71 | 0.34 |
| α-Syn 35-43 + α-Syn 61-80 | 8/21 | 0.93 | [0.83 – 1.00] | 1 | 0.81 | 0.81 |
| α-Syn 35-43 + α-Syn 81-96 | 8/21 | 0.82 | [0.65 – 0.99] | 1 | 0.57 | 0.57 |
| α-Syn 61-80 + α-Syn 81-96 | 8/21 | 0.74 | [0.54 – 0.94] | 0.88 | 0.67 | 0.54 |

# **R script Figure 1**

# Load libraries

library(ggdist)

library(tidyquant)

library(tidyverse)

library(ggpubr)

library(rstatix)

# Define the list of variables

variables <- c("EGVVAAAEK", "QGVAEAAGK", "EGVLYVGSK", "EQVTNVGGAVVTGVTAVAQK", "TVEGAGSIAAATGFVK", "Synaverage", "MSD")

# Define display names for each variable

display_names <- c(

EGVVAAAEK = "a-Syn 13-21",

QGVAEAAGK = "a-Syn 24-32",

EGVLYVGSK = "a-Syn 35-43",

EQVTNVGGAVVTGVTAVAQK = "a-Syn 61-80",

TVEGAGSIAAATGFVK = "a-Syn 81-96",

Synaverage = "a-Syn combined",

MSD = "a-Synuclein (ELISA)"

)

# Load and prepare data with custom Group names

plot_data <- Alpha_syn_data %>%

filter(DG %in% c('CTRL', 'PD', 'MSA', 'DLB')) %>%

mutate(DG = factor(DG, levels = c('CTRL', 'PD', 'MSA', 'DLB')))

# Define colors for the groups

financial_colors <- c("#004b55", "#8e3b46", "#4b0082", "#dba800")

# Custom theme function

theme_tq <- function() {

theme_classic() +

theme(

axis.title = element_text(size = 10),

axis.text = element_text(size = 8)

)

}

# Initialize an empty list to store individual plots

plot_list <- list()

# Loop over each variable and create a plot

for (var in variables) {

# Get the display name for the current variable

display_name <- display_names[var]

# Log-transform MSD only for statistical analysis (not for plotting)

plot_data_stat <- plot_data

if (var == "MSD") {

plot_data_stat <- plot_data_stat %>%

mutate(!!sym(var) := log10(!!sym(var))) # Log-transform for statistical tests

}

# Calculate y-axis extension for consistent space

max_y <- max(plot_data[[var]], na.rm = TRUE)

range_y <- diff(range(plot_data[[var]], na.rm = TRUE))

# Apply additional space specifically for MSD

if (var == "MSD") {

extra_space <- 3 * range_y # Increase extra space for MSD

} else {

extra_space <- 0.3 * range_y

}

# Perform rank-based ANCOVA for the current variable

ancova_result <- plot_data_stat %>%

anova_test(as.formula(paste(var, "~ DG + Age + Sexe")), effect.size = "rank")

# Perform post-hoc pairwise comparisons using Wilcoxon rank-sum test

posthoc_result <- plot_data_stat %>%

pairwise_wilcox_test(as.formula(paste(var, "~ DG")), p.adjust.method = "bonferroni")

# Ensure all comparisons (including non-significant ones) appear

posthoc_result <- posthoc_result %>%

mutate(P_Label = case_when(

p.adj <= 0.0001 ~ "****",

p.adj <= 0.001 ~ "***",

p.adj <= 0.01 ~ "**",

p.adj <= 0.05 ~ "*",

TRUE ~ "ns" # Label non-significant comparisons

))

# Calculate y position for significance labels

num_comparisons <- nrow(posthoc_result)

if (num_comparisons > 0) {

if (var == "MSD") {

y_position_sig <- max_y + (20^(1:num_comparisons / num_comparisons) - 1) * range_y

y_position_sig <- log10(y_position_sig) # Adjust positioning for log scale

} else {

y_position_sig <- max_y + (1:num_comparisons) * 0.1 * range_y

}

posthoc_result <- posthoc_result %>% mutate(y.position = y_position_sig)

}

# Create the plot for the current variable

plot <- plot_data %>%

ggplot(aes_string(x = "DG", y = var, fill = "DG", color = "DG")) +

ggdist::stat_halfeye(

adjust = 0.9,

justification = -0.3,

.width = 0,

alpha = 0.5,

width = 0.6,

point_colour = NA,

height = 0.3

) +

geom_boxplot(

width = 0.20,

outlier.color = NA,

alpha = 0.6,

notch = FALSE,

color = "black"

) +

geom_point(

aes(x = as.numeric(DG) - 0.2),

position = position_jitter(width = 0.05, height = 0),

size = 0.7,

alpha = 1,

show.legend = FALSE

) +

scale_fill_manual(values = financial_colors, name = "") +

scale_color_manual(values = financial_colors, name = "") +

theme_tq() +

labs(

y = ifelse(var == "MSD", paste(display_name, "(pg/mL)"), paste(display_name, "(ratio L/H)")),

fill = ""

) +

ggtitle(display_name) +

theme(plot.title = element_text(size = 10, face = "bold")) +

labs(x = NULL) +

expand_limits(y = max_y + extra_space)

# Apply log scale to y-axis only for MSD visualization

if (var == "MSD") {

plot <- plot + scale_y_log10()

}

# Add significance bars, including "ns" labels

if (nrow(posthoc_result) > 0) {

plot <- plot +

stat_pvalue_manual(

data = posthoc_result,

label = "P_Label",

xmin = "group1",

xmax = "group2",

y.position = "y.position",

inherit.aes = FALSE

)

}

# Add the plot to the list

plot_list[[var]] <- plot

}

# Add uppercase labels (A, B, C, etc.) to subfigures

combined_plot <- ggpubr::ggarrange(

plotlist = plot_list,

ncol = 3,

nrow = ceiling(length(plot_list) / 3),

common.legend = TRUE,

legend = "top",

labels = LETTERS[1:length(plot_list)], # Add A, B, C, ... labels

label.x = 0.02, # Position labels near the top-left

label.y = 1.0,

font.label = list(size = 12) # Adjust label size & boldness

)

# Display the combined plot

print(combined_plot)

ggsave(

filename = "combined_plot.tiff",

plot = combined_plot,

width = 6.6,

height = 8.2,

dpi = 600,

bg = "white"

)

# **R script Figure 2**

library(tidyverse)

library(ggridges)

library(patchwork)

# PART A: Summary Plot (Line Plot with Error Bars)

# Define the acronyms for the peptides

peptide_acronyms <- c(

"EGVVAAAEK" = "α-Syn 13-21",

"QGVAEAAGK" = "α-Syn 24-32",

"EGVLYVGSK" = "α-Syn 35-43",

"EQVTNVGGAVVTGVTAVAQK" = "α-Syn 61-80",

"TVEGAGSIAAATGFVK" = "α-Syn 81-96"

)

# Filter the dataset for the desired disease groups

disease_data <- Alpha_syn_data %>%

filter(DG %in% c("PD", "CTRL", "DLB", "MSA"))

# Transform the data into a long format

long_data <- disease_data %>%

pivot_longer(cols = names(peptide_acronyms),

names_to = "Peptide", values_to = "Abundance")

# Replace peptide sequences with acronyms

long_data <- long_data %>%

mutate(Peptide = factor(Peptide, levels = names(peptide_acronyms), labels = peptide_acronyms))

# Calculate the median, lower quartile, and upper quartile abundance for each peptide and disease group

summary_data <- long_data %>%

group_by(Peptide, DG) %>%

summarize(

Median_Abundance = median(Abundance, na.rm = TRUE),

Lower_Quartile = quantile(Abundance, 0.25, na.rm = TRUE),

Upper_Quartile = quantile(Abundance, 0.75, na.rm = TRUE)

)

# Create the plot for Part A

plot_a <- ggplot(summary_data, aes(x = Peptide, y = Median_Abundance, color = DG, group = DG)) +

geom_point(size = 3) +

geom_line(size = 1.1) +

geom_errorbar(aes(ymin = Lower_Quartile, ymax = Upper_Quartile), width = 0.2, size = 0.7) +

scale_color_manual(values = c("CTRL" = "#004b55", "DLB" = "#8e3b46", "MSA" = "#4b0082", "PD" = "#dba800")) +

theme_minimal() +

labs(

x = "",

y = "Median Abundance",

color = ""

) +

theme(

axis.text.x = element_text(angle = 45, hjust = 1),

legend.position = "top" # Place legend above the plot

)

# PART B: Ridgeline Plots with Legends Above Each Plot

# Define custom gradient start colors for each disease group

group_gradients <- list(

"CTRL" = c("#004b55", "#66B2A8", "#B3E6E1"),

"DLB" = c("#8e3b46", "#D7888E", "#F5B6BA"),

"MSA" = c("#4b0082", "#8151A3", "#C3A6E2"),

"PD" = c("#dba800", "#FFD65C", "#FFF3CD")

)

# Function to create a ridgeline plot for a single group

create_group_plot <- function(group_name) {

ggplot(long_data %>% filter(DG == group_name),

aes(x = Abundance, y = Peptide, fill = after_stat(x))) +

ggridges::geom_density_ridges_gradient(

rel_min_height = 0.01, scale = 3

) +

scale_fill_gradientn(

colors = group_gradients[[group_name]],

name = "Abundance"

) +

theme_minimal() +

labs(

x = "Abundance"

) +

ggridges::theme_ridges(font_size = 13, grid = TRUE) +

theme(

axis.title.y = element_blank(),

strip.text = element_text(size = 12, face = "bold"),

strip.background = element_blank(),

legend.position = "top" # Position legend above each plot

)

}

# Create individual plots for each group

plot_ctrl <- create_group_plot("CTRL")

plot_dlb <- create_group_plot("DLB")

plot_msa <- create_group_plot("MSA")

plot_pd <- create_group_plot("PD")

# Combine the ridgeline plots into a grid for Part B

plot_b <- plot_ctrl + plot_dlb + plot_msa + plot_pd + plot_layout(ncol = 2)

# Wrap Part B into a single plot object to avoid automatic sub-tagging

plot_b_wrapped <- wrap_elements(plot_b)

# Combine Part A and Part B into a single plot

final_plot <- plot_a / plot_b_wrapped +

plot_layout(heights = c(1, 3)) +

plot_annotation(tag_levels = "A", tag_prefix = "", tag_suffix = "",

theme = theme(plot.tag.position = c(0, 1), # Position top-left

plot.tag = element_text(size = 16, face = "bold")))

# Print the final combined plot

print(final_plot)

# Save the final plot as an image file

ggsave(

filename = "combined_plot_with_labels.png",

plot = final_plot,

width = 7,

height = 9,

dpi = 600

)

# **R script Figure 3**

# Load necessary libraries

library(corrplot)

library(tidyverse)

# Define the function to compute the p-value matrix

cor.mtest <- function(mat, ...) {

mat <- as.matrix(mat)

n <- ncol(mat)

p.mat <- matrix(NA, n, n)

diag(p.mat) <- 0

for (i in 1:(n - 1)) {

for (j in (i + 1):n) {

tmp <- cor.test(mat[, i], mat[, j], ...)

p.mat[i, j] <- p.mat[j, i] <- tmp$p.value

}

}

colnames(p.mat) <- rownames(p.mat) <- colnames(mat)

return(p.mat)

}

# Define the function to determine significance labels

get_sig_label <- function(p.value) {

if (p.value < 0.001) {

return("***")

} else if (p.value < 0.01) {

return("**")

} else if (p.value < 0.05) {

return("*")

} else {

return("")

}

}

# Specify variables to include in the plot

variables <- c("EGVVAAAEK", "QGVAEAAGK", "EGVLYVGSK", "EQVTNVGGAVVTGVTAVAQK", "TVEGAGSIAAATGFVK", "MSD")

# Define human-readable display names

display_names <- c(

EGVVAAAEK = "a-Syn 13-21",

QGVAEAAGK = "a-Syn 24-32",

EGVLYVGSK = "a-Syn 35-43",

EQVTNVGGAVVTGVTAVAQK = "a-Syn 61-80",

TVEGAGSIAAATGFVK = "a-Syn 81-96",

MSD = "a-Synuclein (ELISA)"

)

# Manually select a group (change this to "CTRL", "DLB", or "MSA" as needed)

selected_group <- "MSA"

# Filter the dataset for the selected group and variables

filtered_data <- Alpha_syn_data %>%

filter(DG == selected_group) %>%

select(all_of(variables))

# Compute the correlation matrix

M <- cor(filtered_data, use = "pairwise.complete.obs")

# Compute the p-value matrix

p.mat <- cor.mtest(filtered_data)

# Rename variables for the plot using display names

colnames(M) <- rownames(M) <- display_names[names(display_names) %in% colnames(M)]

colnames(p.mat) <- rownames(p.mat) <- display_names[names(display_names) %in% colnames(p.mat)]

# ---- SAVE PLOT AS TIFF (600 DPI) ----

tiff("correlation_plot.tiff", width=6, height=6, units="in", res=600) # Set TIFF properties

# Plot the correlation matrix with significance stars

corrplot(M, method="circle", type="upper",

tl.col="black", tl.srt=45, tl.cex=0.8, # Adjust axis labels

cl.cex=0.8, cl.align.text = "l", # Adjust color legend

mar=c(0, 0, 0, 0)) # Remove margins

# Add significance stars manually

n <- ncol(p.mat)

for (i in 1:(n-1)) {

for (j in (i+1):n) {

label <- get_sig_label(p.mat[i, j])

if (label != "") {

text(j, n-i+1, label, cex=1.3, col="white") # Add stars

}

}

}

dev.off() # Close the TIFF device

# --------------------------------------

# **R script Figure 4**

library(pROC)

library(ggplot2)

library(tidyquant)

# Assuming your data frame is named "Alpha_syn_data"

data <- Alpha_syn_data

# Filter out non-PD samples that are not MSA

data_filtered <- subset(data, PD == 1 | (PD == 0 & MSA == 1))

# Specify the names of your variables

variable_names <- c("EGVVAAAEK", "QGVAEAAGK", "EGVLYVGSK", "TVEGAGSIAAATGFVK", "EQVTNVGGAVVTGVTAVAQK")

# Outcome variable

outcome_variable <- "PD"

# Initialize storage

auc_list <- list()

model_list <- list()

var_list <- list()

auc_ci_list <- list()

# Mapping variable names to display names

display_names <- c(

EGVVAAAEK = "aSyn 13-21",

QGVAEAAGK = "aSyn 24-32",

EGVLYVGSK = "aSyn 35-43",

TVEGAGSIAAATGFVK = "aSyn 81-96",

EQVTNVGGAVVTGVTAVAQK = "aSyn 61-80"

)

# Loop through variable combinations (up to 2 at a time)

for (k in 1:2) {

combinations <- combn(variable_names, k, simplify = FALSE)

for (combination in combinations) {

current_vars <- combination

data_subset <- data_filtered[complete.cases(data_filtered[, c(current_vars, outcome_variable)]), ]

if (nrow(data_subset) > 0) {

formula <- as.formula(paste(outcome_variable, "~", paste(current_vars, collapse = "+")))

tryCatch({

model <- glm(formula, data = data_subset, family = binomial(link = "logit"))

predicted_probabilities <- predict(model, newdata = data_subset, type = "response")

roc_obj <- roc(response = data_subset[[outcome_variable]], predictor = predicted_probabilities)

# Store AUC, model, vars

auc <- auc(roc_obj)

auc_list[[length(auc_list) + 1]] <- auc

model_list[[length(model_list) + 1]] <- model

var_list[[length(var_list) + 1]] <- current_vars

# 95% CI

auc_ci <- ci(roc_obj, of = "auc", conf.level = 0.95)

auc_ci_list[[length(auc_ci_list) + 1]] <- auc_ci

cat("Successfully processed combination: ", paste(current_vars, collapse = ", "), "\n")

}, error = function(e) {

cat("Error with combination: ", paste(current_vars, collapse = ", "), ". Error message: ", e$message, "\n")

})

} else {

cat("Combination skipped due to insufficient data: ", paste(current_vars, collapse = ", "), "\n")

}

}

}

# Identify top 5 AUCs

top_auc_indices <- order(unlist(auc_list), decreasing = TRUE)[1:5]

# Apply display names to variables

var_list_display_names <- lapply(var_list, function(vars) sapply(vars, function(var) display_names[var]))

# Generate legend labels

legend_labels <- mapply(function(vars, auc, ci) {

paste(paste(vars, collapse = " + "),

sprintf("; AUC= %.2f", auc),

sprintf("95%%CI= [%.2f-%.2f]", ci[1], ci[3]))

}, var_list_display_names[top_auc_indices], auc_list[top_auc_indices], auc_ci_list[top_auc_indices], SIMPLIFY = FALSE)

# Custom color palette

custom_palette <- c("#364B9A", "#E67E22", "#009E73", "#F0E442", "#8E44AD")

# Build plot data

plot_data <- data.frame()

for (i in top_auc_indices) {

specific_vars <- var_list[[i]]

data_subset <- data_filtered[complete.cases(data_filtered[, c(specific_vars, outcome_variable)]), ]

predicted_probabilities <- predict(model_list[[i]], newdata = data_subset, type = "response")

roc_obj <- roc(response = data_subset[[outcome_variable]], predictor = predicted_probabilities)

df <- coords(roc_obj, "all", transpose = FALSE)

df$Group <- paste0("ROC ", i)

plot_data <- rbind(plot_data, df)

}

# Assign correct group order

plot_data$Group <- factor(plot_data$Group, levels = paste0("ROC ", top_auc_indices))

# Line styles

line_styles <- c("solid", "dashed", "dotted", "dotdash", "twodash")

# Plotting

plot <- ggplot(plot_data, aes(x = 1 - specificity, y = sensitivity, color = Group, linetype = Group)) +

geom_abline(intercept = 0, slope = 1, linetype = "dashed", color = "gray40") + # Diagonal line

geom_path(size = 1.2) +

scale_color_manual(labels = legend_labels, values = custom_palette) +

scale_linetype_manual(values = line_styles) +

theme_tq() +

labs(

x = "1 - Specificity",

y = "Sensitivity"

) +

guides(

color = guide_legend(title = "PD vs MSA", override.aes = list(

linetype = line_styles

)),

linetype = "none"

) +

theme(

legend.text = element_text(size = 8),

legend.position = c(1, 0),

legend.justification = c(1, 0),

legend.title = element_text(size = 11, face = "bold"),

legend.background = element_rect(fill = "white"),

legend.box.margin = margin(5, 5, 5, 5),

legend.margin = margin(5, 5, 5, 5),

legend.key.size = unit(1, "lines"),

plot.title = element_text(size = 16, face = "bold", hjust = 0.5),

axis.title = element_text(size = 12),

axis.text = element_text(size = 10),

panel.grid.minor = element_blank(),

panel.grid.major.x = element_blank(),

panel.grid.major.y = element_blank(),

plot.margin = margin(10, 10, 10, 10)

) +

coord_fixed(ratio = 1)

# Print and save

print(plot)

ggsave(

filename = "combined_plot.tiff",

plot = plot,

width = 5,

height = 5,

dpi = 600

)
